# A systematic review of the impact of *Porphyromonas gingivalis* on foam cell formation: Implications for the role of periodontitis in atherosclerosis

**DOI:** 10.1186/s12903-023-03183-9

**Published:** 2023-07-13

**Authors:** Saeed Afzoon, Mohammad Amin Amiri, Mostafa Mohebbi, Shahram Hamedani, Nima Farshidfar

**Affiliations:** 1 Student Research Committee, Shiraz University of Medical Sciences, Shiraz, Iran; 2 Oral and Dental Disease Research Center, School of Dentistry, Shiraz University of Medical Sciences, Shiraz, Iran; 3 Orthodontic Research Center, School of Dentistry, Shiraz University of Medical Sciences, Shiraz, Iran

**Keywords:** Periodontitis, Chronic Periodontitis, Porphyromonas Gingivalis, Atherosclerosis, Foam cells

## Abstract

**Background:**

The current literature suggests the significant role of foam cells in the initiation of atherosclerosis through the formation of a necrotic core in atherosclerotic plaques. Moreover, an important periodontal pathogen called *Porphyromonas gingivalis (P. gingivalis)* is indicated to play a significant role in this regard. Thus, the aim of this systematic review was to comprehensively study the pathways by which *P. gingivalis* as a prominent bacterial species in periodontal disease, can induce foam cells that would initiate the process of atherosclerosis formation.

**Methods:**

An electronic search was undertaken in three databases (Pubmed, Scopus, and Web of Science) to identify the studies published from January 2000 until March 2023. The risk of bias in each study was also assessed using the QUIN risk of bias assessment tool.

**Results:**

After the completion of the screening process, 11 in-vitro studies met the inclusion criteria and were included for further assessments. Nine of these studies represented a medium risk of bias, while the other two had a high risk of bias. All of the studies have reported that *P. gingivalis* can significantly induce foam cell formation by infecting the macrophages and induction of oxidized low-density lipoprotein (oxLDL) uptake. This process is activated through various mediators and pathways. The most important factors in this regard are the lipopolysaccharide of *P. gingivalis* and its outer membrane vesicles, as well as the changes in the expression rate of transmembrane lipid transportation channels, including transient receptor potential channel of the vanilloid subfamily 4 (TRPV4), lysosomal integral protein 2 (LIMP2), CD36, etc. The identified molecular pathways involved in this process include but are not limited to NF-κB, ERK1/2, p65.

**Conclusion:**

Based on the results of this study, it can be concluded that *P. gingivalis* can effectively promote foam cell formation through various pathogenic elements and this bacterial species can affect the expression rate of various genes and the function of specific receptors in the cellular and lysosomal membranes. However, due to the moderate to high level of risk of bias among the studies, further studies are required in this regard.

## Background

Periodontal diseases are inflammatory conditions which affect the periodontal tissue and consequently result in soft tissue recession, bone loss, tooth loss, and mild elevation of systemic inflammatory factors [1–5]. Based on the current evidence, 20–50% of the global health population are affected by periodontal diseases; therefore, its high prevalence makes it an important public health issue [6, 7]. Approximately 700 species of bacteria are identified in the oral cavity and it is proposed that the interaction of periodontal pathogens and host response can lead to periodontal diseases [8, 9].

Many pathogens are associated with the development of periodontitis among which *Porphyromonas gingivalis (P. gingivalis)* acts as a critical factor in the progression of periodontal pathologies [10, 11]. This process is mediated through the modified expression of multiple growth factors in periodontal tissues [12–14]. This bacterial species produces different virulence factors which could induce and sustain systemic inflammation [15]. *P. gingivalis* is also able to degenerate the tissue and cause local and systemic pathologies [15]. Recent studies have indicated a possible association of *P. gingivalis* with different systemic diseases, such as cardiovascular, cerebral, pulmonary, digestive, bone, and perinatal disease [16]. Among all the mentioned systemic conditions, one of the most noticeable diseases with high cardiovascular complications is atherosclerosis [17].

Atherosclerosis is a lipid-driven inflammatory disease caused by dysregulation of lipid metabolism resulting in the accumulation of lipid droplets in the matrix beneath an endothelial layer of arteries [18, 19]. This vascular pathology is one the main causes of cardiovascular diseases, heart failure, stroke, and myocardial infarction [20, 21]. It can also lead to vascular complications, such as coronary artery disease, carotid artery disease, and peripheral arterial disease [22]. One of the major processes playing a crucial role in the occurrence of atherosclerosis is foam cell formation [23]. The increase in cholesterol level makes the arteries more permeable which results in monocytes infiltration to the sub-endothelial layer where they convert into macrophages [15, 19, 24, 25]. Excessive uptake of lipids and oxidized low-density lipoprotein (oxLDL) stored in macrophage cytoplasm eventually changes the macrophage metabolism [23]. Consequently, immoderate accumulation of oxLDL in macrophage cytoplasm exceeds the capacity of macrophage to continue normal lipid metabolism [26–28]. This process eventually results in macrophage apoptosis and gradual formation of foamy cells [26–28].

Studies have declared a marked correlation between atherosclerosis and periodontitis [29–32]. Therefore, the process of foam cell formation, as one of the major mechanisms of atherosclerosis, could be affected by the presence of *P. gingivalis* in patients who have developed periodontitis [33]. It has been reported that *P. gingivalis* has been found in arterial plaque in humans and mice [30, 34]. The abilities of this bacteria to circumvent the immune system could contribute to the induction and progression of atherosclerosis [30, 31]. Besides, it was found that *P. gingivalis* is able to accelerate lipid peroxidation and the progression of atherosclerosis in the presence of oxLDL [32]. Several studies have indicated that this process is carried out through the infection of macrophages in the arterial intima layer with *P. gingivalis* [35–37]. In order to obtain a comprehensive insight into this process, we have performed a systematic review of the different mechanisms by which *P. gingivalis* can induce foam cell formation.

## Materials and methods

### Protocol development

This systematic review follows the guidelines recommended by The Preferred Reporting Items for Systematic reviews and Meta-Analyses (PRISMA) statement 2020 [38, 39].

### Information sources and search strategy

The PubMed, Scopus, and Web of Science databases were searched to identify the articles reporting the association of *P. gingivalis* with foam cell formation from January 1, 2000, until March 12, 2023. In addition, independently, a manual search was conducted by two authors, in an act of perusing reference lists of included papers to find further studies associated with the topic.

We searched the mentioned databases using the following combination of free-text terms:

**(**Foam Cells **OR** Macrophages**) AND (**Porphyromonas gingivalis **OR** Porphyromonas **OR** Bacteroides gingivalis).

### Eligibility criteria

Table 1 illustrates the eligibility criteria for the aspects of participants, intervention, comparison, outcomes, and study design (PICOS). All in vitro studies investigating the possible effect of *P. gingivalis* on foam cell formation were included in this review. The quantity of foam cell formation had to be explored in the presence of *P. gingivalis* in comparison to the control group in which no *P. gingivalis* was present. Studies evaluating the effect of *P. gingivalis* in the absence of the control group were excluded. Additionally, studies other than in-vitro studies such as ex-vivo studies, in-vivo studies, etc. were excluded. Studies in languages other than English or Persian were excluded from our review considering the linguistic competency of the research team.

**Table 1:** Representation of the PICOS of the systematic review

PICOS	Inclusion Criteria	Exclusion Criteria
**Population**	Studies assessing cultured macrophage cells	Studies assessing cells other than macrophages
**Intervention**	Studies evaluating the effect of *P. gingivalis*	Studies evaluating the effect of bacteria other than *P. gingivalis*
**Comparison**	Studies evaluating a group of macrophages without exposure to any bacterial species	-
**Outcome**	Studies assessing the rate of foam cell formation from macrophage cells	-
**Study Design**	In-vitro studies	Case reports, narrative reviews, systematic reviews with or without meta-analysis, letters to the editors, short communications, in-vivo studies, ex-vivo studies, animal studies, and non-comparative studies.

### Study selection

Based on the eligibility criteria, the authors (MAA, SA, and MM) screened the title and abstract of the retrieved articles independently. Furthermore, the retrieved articles were scrutinized for any possible predatory publication. In the case of disagreement, all the aforementioned researchers discussed the matter with other authors (SH and NF) to reach an agreement. The full texts of the selected articles were obtained, and studies meeting the inclusion criteria were included in our systematic review.

### Data collection and data items

In a customized data extraction manner, the name of the authors, the year of publications, the type of evaluations, the evaluation methods, the main outcomes, the key molecular elements, and the mechanism of action were extracted.

### Risk of bias assessment

In this systematic review, we used a novel risk of bias assessment tool named QUIN tool which was recently introduced by Sheth et al. in 2022 [40]. QUIN tool was mainly introduced for the evaluation of the risk of biases within the in-vitro studies in the field of dentistry. This risk of bias tool contains 12 criteria, and each of them, as represented in Table 2, can be scored as either 2 (adequately specified), 1 (poorly specified), 0 (not specified), or NA (not applicable). The total score is also estimated by the following formula:

$$\text{Final score}=\frac{Total score\times 100}{Number of applicable criteria\times 2}$$

**Table 2:** Assessment of risk of bias in each study using the QUIN tool

Criteria number	Criteria	Qi et al. (2003) [35]	Kuramitsu et al. (2003) [45]	Miyakawa et al. (2004) [36]	Giacona et al. (2004) [37]	Shaik-Dasthagirisaheb et al. (2013) [42]	Li et al. (2013) [47]	Shaik-Dasthagirisheb et al. (2016) [43]	Liang et al. (2016) [46]	Kim et al. (2018) [48]	Gupta et al. (2019) [44]	Yang et al. (2020) [41]
1	Clearly stated aims/objectives	2	1	2	2	2	2	1	2	2	2	2
2	Detailed explanation of sample size calculation	NA	NA	NA	NA	NA	NA	NA	NA	NA	NA	NA
3	Detailed explanation of the sampling technique	NA	NA	NA	2	2	NA	2	2	2	2	2
4	Details of the comparison group	2	1	1	1	2	1	1	2	2	2	1
5	Detailed explanation of the methodology	2	2	2	2	2	2	2	2	2	2	2
6	Operator details	0	0	0	0	0	0	0	0	0	0	0
7	Randomization	NA	NA	NA	NA	NA	NA	NA	NA	NA	NA	NA
8	Method of measurement of outcome	2	2	2	2	2	2	2	2	2	2	2
9	Outcome assessor details	0	0	0	0	0	0	0	0	0	0	0
10	Blinding	0	0	0	0	0	0	0	0	0	0	0
11	Statistical analysis	0	0	0	2	2	2	0	2	2	2	2
12	Presentation of results	2	2	2	2	2	2	0	2	2	2	2
Total score		55.55%	44.44%	50%	65%	70%	61.11%	40%	70%	70%	70%	65%
Risk of bias		Medium	High	Medium	Medium	Medium	Medium	High	Medium	Medium	Medium	Medium

A total score above 70% indicates a low risk of bias, a total score between 50 and 70% suggests a medium risk of bias, and a total score less than 50% represents a high risk of bias in the study.

## Results

### Study selection

**Fig. 1:**
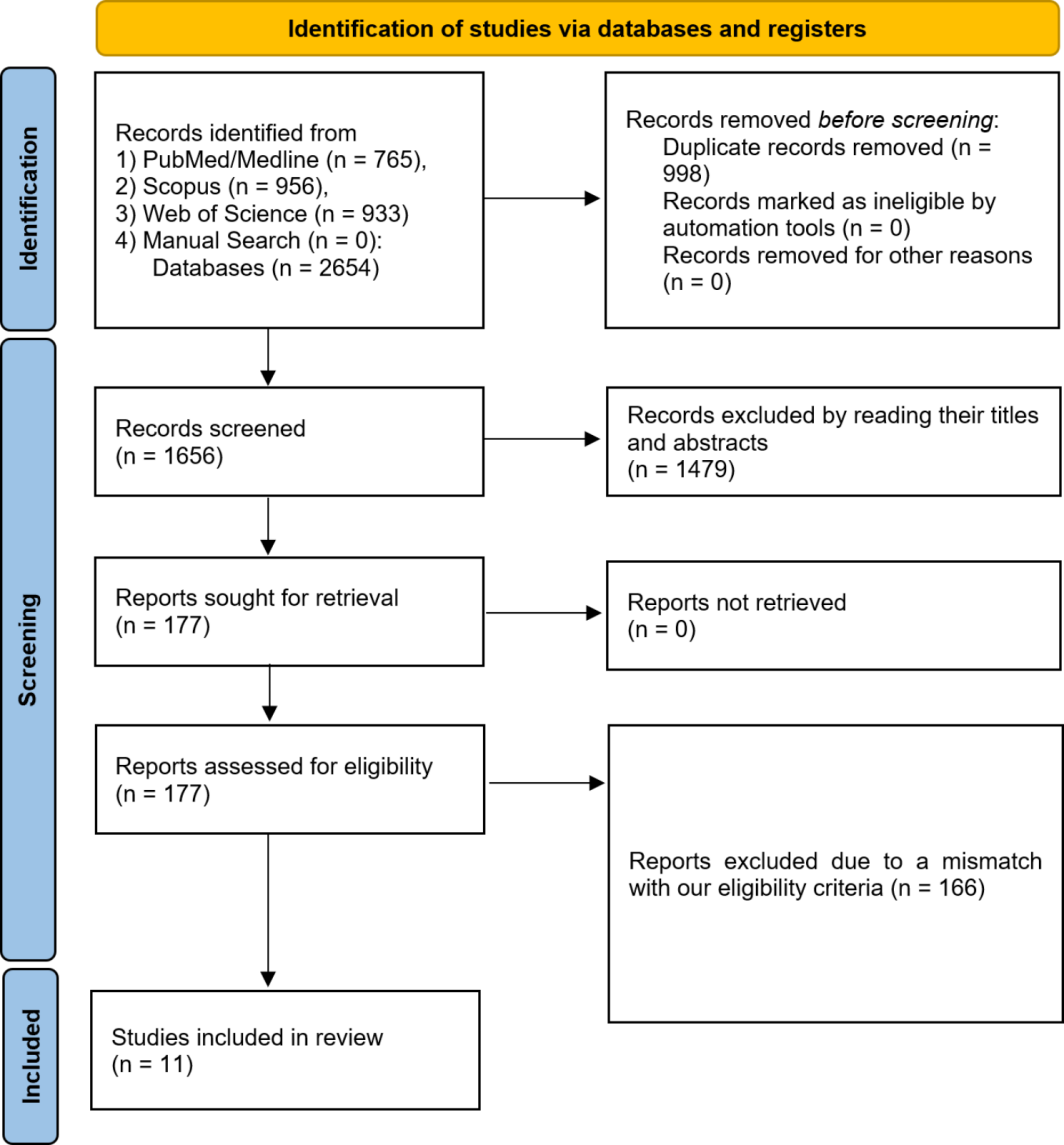
PRISMA chart 2020 representing the screening process undertaken in this study

The initial search of three databases identified 2654 studies in total. After duplicate removal, 1656 abstracts and titles remained and underwent screening. A total of 1479 papers were excluded due to a mismatch with our search criteria and 177 articles were retained for eligibility assessment and full-text review among which 166 were screened by title and abstract and didn’t meet the eligibility criteria mentioned in Table 1. Finally, 11 original articles were included in this systematic review. The PRISMA chart below briefly represents the aforementioned process (Fig. 1).

### Study characteristics

There were 11 studies eligible for the systemic review. Table 3 presents detailed individual characteristics, including study groups, type, and method of evaluation, and the outcomes of each study.

**Table 3:** Summary of included studies

Author (Year)	Type(s) of Evaluation	Method(s)	Main Outcome(s)	References
Yang et al. (2020)	Foam cell formation	Oil Red O staining	The knockdown of *limp2* reduces the rate of foam cell formation and enhances cholesterol export. The interaction of LIMP2 and caveolin-1 (CAV1) in the lysosome of macrophages may play a key role in this regard.	[41]
	Cathepsin L activity	Magic red cathepsin L assay		
	RNA sequencing	RT-PCR		
	Protein detection	Western blot		
	Protein detection	Co-immunoprecipitation		
Gupta et al. (2019)	Binding and uptake of oxLDL	Fluorescence intensity microscopy	TRPV4 plays a key part in foam cell formation and inflammatory genes upregulation, which is subsequent to LDL oxidation. This process was also induced by *P. gingivalis* LPS.	[44]
	Foam cell formation	Oil Red O Staining		
	Expression levels of TRPV4, actin, or CD36	Immunoblot and immunofluorescence assay		
Kim et al. (2018)	Oxidation extent of HDL or LDL	TBARS assay	HDL incubated with *P. gingivalis* showed significantly higher oxidation levels and TNF- α production. *P. gingivalis* induces HDL oxidation, by proinflammatory response in interaction with macrophages.	[48]
	TNF-α	ELISA		
	The activity of MMPs, and Gelatinase	Electrophoresis, Gelatin zymography		
	Foam cells	Oil Red O staining		
Liang et al. (2016)	Foam cell formation	Oil Red O staining	*P. gingivalis* can induce foam cell formation through the upregulation of CD36 expression in macrophages. CD36 expression in the presence of *P. gingivalis* is mediated by NF-κB, ERK1/2, and p65.	[46]
	NF-κB activity	RT-PCR		
	CD36 protein levels	Western blot		
	NF-κB activity	Luciferase reporter assay		
	The interaction of NF-κB and CD36 promoters	EMSA		
	The interaction of NF-κB and CD36 promoters	Chromatin immunoprecipitation assay		
	CD36 protein levels	Flow cytometry		
Shaik-Dasthagirisheb et al. (2016)	Foam cell formation	Oil Red O Staining	Both *P. gingivalis* and *C. pneumonia* can induce foam cell formation in macrophages.	[43]
	Lipid peroxidation	TBARS assay for level of oxidized LDL	*P. gingivalis* enhances LDL oxidation while no statistical difference was reported between the species.	
	Inflammatory cytokines secretion	ELISA	Both *P. gingivalis* and *C. pneumonia* enhance TNF-α and IL-6 secretion from LDL-treated macrophages.	
	Gene expression	PCR	Despite the differences between *P. gingivalis* and *C. pneumonia*, they indicate a similar pattern in activation and down-regulation of genes in macrophages.	
Li et al. (2013)	Foam cell formation	Oil Red O staining	*P. gingivalis* LPS can promote foam cell formation in ox-LDL-treated macrophages. *P. gingivalis* LPS could enhance CD36 mRNA expression which acts as a mediator receptor for lipid uptake and decrease the cholesterol efflux by down-regulation of ABCA1.	[47]
	Cholesterol efflux	Cholesterol efflux assay		
	Expression of ABCA1, CD36	RT-PCR		
	HO-shRNA level	Western blot		
Shaik-Dasthagirisaheb et al. (2013)	Foam cell formation	Oil Red O staining	The sole addition of *P. gingivalis* to macrophages could enhance foam cell formation; however, the sole addition of LDL did not demonstrate the same effect. Moreover, heat-killed *P. gingivalis* had a similar effect on foam cell formation compared to alive *P. gingivalis*, regardless of the presence or the absence of LDL.	[42]
	MyD88 and lps2 gene’s role in foam cell formation	Oil Red O staining	In both concurrent and uncoupled methods, MyD88 gene knockout demonstrated substantial reductions in a number of foam cells compared to the naïve types. However, in the presence of LDL lps2-knockout mice formed foam cells similar to naïve types.	
	Effect of *P. gingivalis* dose on Foam cell formation	Oil Red O staining	Enhanced concentrations of *P. gingivalis* (MOI of 1, 10, and 100), regardless of the concurrent or uncoupled LDL treatment, elicited a greater percentage of foam cells	
	Effect of LDL on the production of inflammatory cytokines	ELISA	The elevated levels of LDL significantly decrease the pro-inflammatory cytokine production by macrophages cultured with *P. gingivalis.*	
Giacona et al. (2004)	Foam cell formation	Oil Red O staining	The results indicate the higher effect of naïve *P.g* compared to fimbria-deficient *P. gingivalis* to induce foam cell formation.	[37]
	Recovery of viable *P. gingivalis* from antibiotic-treated macrophages	Antibiotic protection assay	Recovery of naïve *P. gingivalis* species was significantly higher than the fimbria-deficient ones.	
	Uptake of *P. gingivalis* by macrophages	Transmission electron microscopy	The naïve *P. gingivalis* types are more capable in adhering and entering the macrophage cells than the fimbria-deficient ones.	
Miyakawa et al. (2004)	Foam cell formation by aggregated LDL	Oil Red O staining	*P. gingivalis* and its OMVs induce dose-dependent LDL aggregation and eventually foam cell formation, which is in part performed by the proteolysis of apo B-100 protein that is involved in the transportation of LDL.	[36]
	LDL aggregation	Transmission electron microscope		
		SDS–PAGE and western blotting		
	LDL modification	Relative electrophoresis mobility (REM) shift assays		
Kuramitso et al. (2003)	Foam cell formation	Oil Red O staining	*P. gingivalis* promotes foam cell formation which the most important element in this regard seems to be the *P. gingivalis* LPS. Moreover, *P. gingivalis* can induce MCP-1 secretion in endothelial cells.	[45]
	MCP-1	ELISA		
Qi et al. (2003)	Effect of *P. g* foam cell formation	Oil Red O staining	*P. gingivalis* LPS alone cannot induce foam cell formation by itself. The presence of *P. gingivalis* and its’ OMVs can modify LDL and induce foam cell formation.	[35]
	Effect of OMV on foam cell formation			
	Effect of LPS on foam cell formation			
	Effect of LDL-uptake on foam cell formation	Fluorescence imaging of LDL binding to macrophages		
	LDL modification by *P. gingivalis* during foam cell formation	Agarose gel electrophoresis		
	LDL peroxidation induced by *P. gingivalis*	TBARS assay		

**Abbreviations**: ABCA1: ATP-binding cassette transporter A1, ELISA: Enzyme-linked immunosorbent assay, LDL: Low-density lipoprotein, MCP-1: Monocyte chemoattractant protein-1, MMP: matrix metalloproteinase, MOI: Multiciplity of Infection, P. gingivalis: Porphyromonas gingivalis, OMV: Outer Membrane Vesicles, oxLDL: oxidized low-density lipoprotein, SDS-PAGE: Sodium Dodecyl Sulfate-Polyacrylamide Gel Electrophoresis, TBARS: Thiobarbituric acid-reactive substances assay

Out of 11 studies, four studies used murine bone marrow-derived macrophages [41–44], two studies used J-774 murine macrophage-like cells [35, 36, 45], one study used peritoneal macrophages [46] and three of them used THP-1-derived macrophages/monocytes [37, 47, 48]. Eight out of 11 studies used LDL [35–37, 42–45, 48] or oxLDL [41, 46–48] and only one study used high-density lipoprotein (HDL) [48].

Furthermore, four studies used *P. gingivalis* lipopolysaccharide (LPS) [35, 44, 45, 47] and others used *P. gingivalis* [35–37, 41, 42, 45, 46, 48]. All the studies evaluated foam cell formation by Oil Red O staining [28–38]. Three studies assessed cholesterol accumulation due to *P. gingivalis* [35, 41, 47], and three studies evaluated inflammatory cytokines formation [42, 43, 48].

### Results of individual studies

All studies showed foam cell formation as *P. gingivalis* or *P. gingivalis* LPS were used [28–38]. Eight studies evaluated foam cell formation in the presence of LDL and showed its necessity for the formation of foam cells promoted by *P. gingivalis* [35–37, 42–45, 48]. Two studies showed increased LDL oxidization induced by *P. gingivalis* [35, 43] and one study reported the same results for HDL [48]. Three studies showed that *P. gingivalis* increases cholesterol accumulation [35, 41, 47]. On the other hand, three studies that evaluated inflammatory cytokines levels showed *P. gingivalis*-induced promotion of inflammatory cytokines [42, 43, 48]. Table 3 illustrates the variables of each study, as well as their methods of evaluation and main outcomes. Moreover, Table 4 was added to emphasize the type of pathogenic element on which each study has focused as well as their mechanism of action.

**Table 4:** Review of the key molecular elements and their mechanism of action used by P. gingivalis to induce foam cell formation

Authors (Year)	Key molecular element	Mechanism of Action	References
Yang et al. (2020)	LIMP2	*P. gingivalis* induces foam cell formation via NF-κB and JNK pathways, which enhance the expression of LIMP2, caveolin-1 (CAV-1), and their interactions.	[41]
Gupta et al. (2019)	TRPV4	TRPV4 can regulate oxLDL uptake in macrophages and this mechanosensitive channel is sensitive to the extracellular matrix stiffness induced by *P. gingivalis* LPS.	[44]
Kim et al. (2018)	HDL	*P. gingivalis* can induce HDL oxidation, which prevents its athero-protective effects and promotes athero-inductive effects by eliciting pro-inflammatory cytokines secretion.	[48]
Liang et al. (2016)	CD36, NF-κB, ERK1/2, and p65	The *P. gingivalis* infection can cause CD36 upregulation through the pathways mediated by NF-κB, ERK1/2, and p65.	[46]
Shaik-Dasthagirisaheb et al. (2016)	Modification of genes subsequent in macrophage-infected *P. gingivalis*	*P. gingivalis* can up-regulate and down-regulate the genes involved in lipid uptake and efflux, respectively. *P. gingivalis* can also enhance the expression of genes associated with inflammatory biomarkers, cell adhesion, and ECM modification.	[43]
Li et al. (2013)	*P. gingivalis* LPS, CD36, ABCA-1, calpain, HO-1	*P. gingivalis* LPS induces foam cell formation through HO-1 expression, which results in the activation of the cJun/AP-1 pathway that can promote upregulation of CD36 and downregulation of ABCA-1via upregulation of calpain activity.	[47]
Shaik-Dasthagirisaheb et al. (2013)	*P. gingivalis* LPS, Myeloid differentiation factor 88 (MyD88)	*P. gingivalis* LPS can induce foam cell formation, regardless of the presence or the absence of LDL. Moreover, the knockout of the MyD88 gene can markedly reduce foam cell formation.	[42]
Miyakawa et al. (2004)	OMV	*P. gingivalis* and its OMVs could induce LDL aggregation in a dose-dependent manner by proteolysis of apo B-100 protein and modification of LDL to induce higher mobility of the final LDLs.	[36]
Giacona et al. (2004)	*P. gingivalis* fimbria	The major fimbria of *P. gingivalis* plays a key role in inducing foam cell formation and *P. gingivalis* invasion into the macrophage cells. Moreover, the major fimbria enhances the recovery of *P. gingivalis* in the presence of antibiotics.	[37]
Kuramitso et al. (2003)	*P. gingivalis* fimbria, *P. gingivalis* LPS, MCP-1	The induction of MCP-1 secretion from the endothelial cells, caused by *P. gingivalis*, can attract more monocytes to the site and accelerate the process of foam cell formation and eventually, atherosclerosis.	[45]
Qi et al. (2003)	*P. gingivalis* LPS, OMV	Induction of cholesterol binding and intake by macrophages	[35]

**Abbreviations**: ECM: Extra-cellular matrix, HDL: High-density lipoprotein, HO-1: heme oxygenase-1, LIMP2: lysosomal integral protein 2, P. gingivalis: Porphyromonas gingivalis, OMV: Outer Membrane Vesicles, TRPV4: Transient receptor potential channel of the vanilloid subfamily 4

### Risk of bias assessment

Out of 11 studies included in this study, nine studies represented a medium risk of bias [35–37, 41, 42, 44, 46–48] while two studies had a high risk of bias [43, 45] **(**Table 2).

## Discussion

**Fig. 2:**
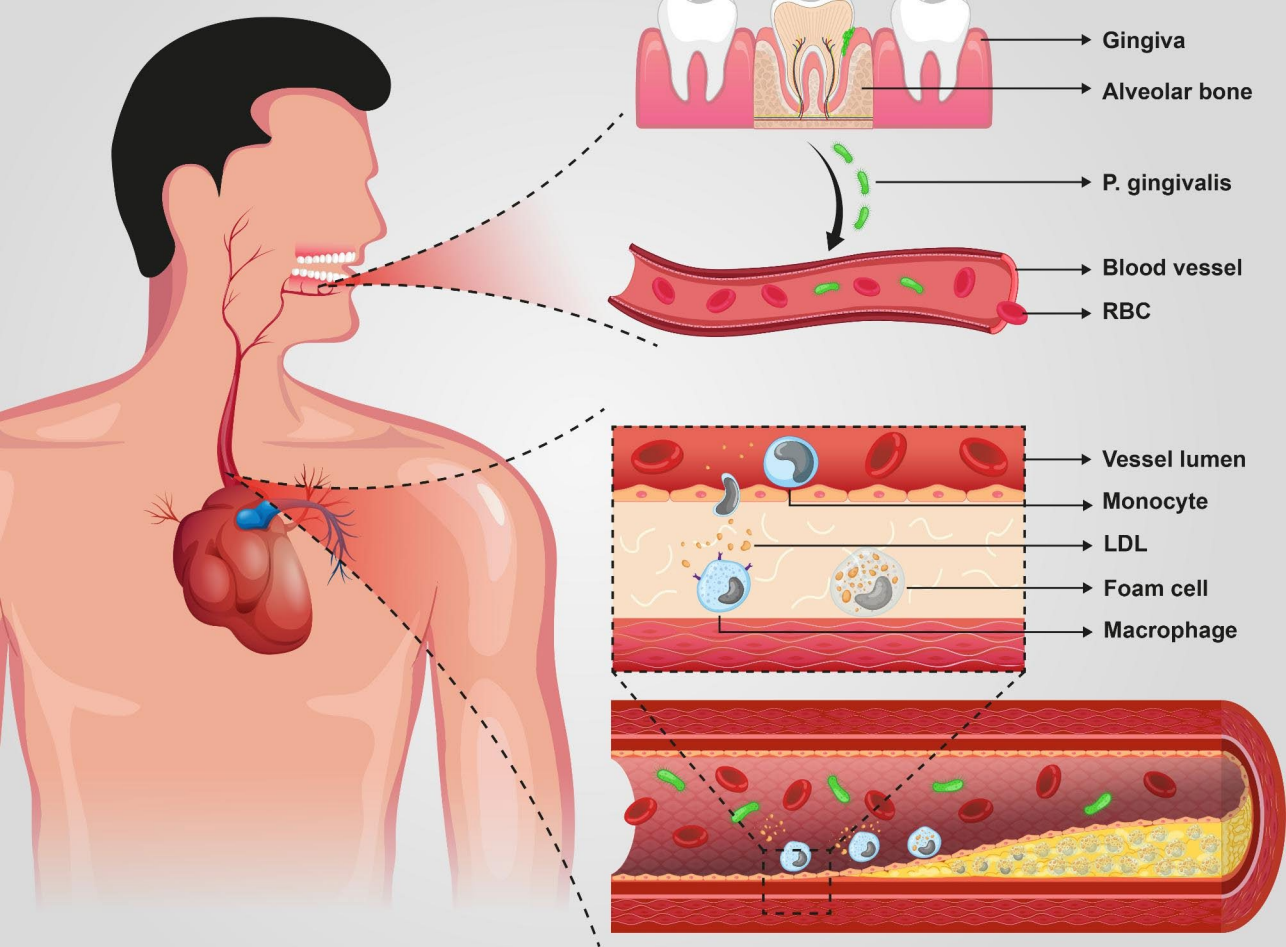
Illustrates the process by which *P. gingivalis* can play a role in foam cell formation. The infection caused by periodontopathogens, especially *P. gingivalis* destructs periodontal tissue. The progression of this disease leads to the infiltration of *P. gingivalis* into blood vessels which by entering into the blood vessels intima, it can affect the macrophages in that layer and induce LDL endocytosis. This process eventually leads to foam cell formation

Based on the results of this study, *P. gingivalis* plays an imperative role in macrophage foam cell formation. This process is clearly described in Fig. 2. Although the exact mechanisms through which this process takes place are not thoroughly uncovered, several pathways and molecules are suggested to have a significant role in this process [43, 44, 46, 47, 49–51].

The first and foremost pathway which was suggested by several studies is the scavenger receptors [41, 43, 47]. The macrophage scavenger receptors attach to the modified lipoproteins and enhance cellular cholesterol accumulation [52]. In this regard, CD36 is shown to increase in macrophage after exposure to *P. gingivalis* [52]. In a study by Li et al. [47], the *P. gingivalis* LPS could induce LDL accumulation and inhibit cholesterol efflux during the process of foam cell formation. In more detail, during the macrophage foam cell formation process, *P. gingivalis* LPS promotes CD36 mRNA and its protein expression, as well as inhibiting ATP–binding cassette transporter A1 (ABCA1) [47]. The *P. gingivalis* LPS-induced CD36 expression and ABCA1 inhibition are mediated through the activation of c–Jun-AP/1 and increased calpain activity [47]. Moreover, c–Jun-AP/1 is found to be the key transcriptional factor in *P. gingivalis* LPS–induced CD36 upregulation [47]. It is worth mentioning that LPS of *P. gingivalis* did not seem to have any effect on scavenger receptor A (SRA), scavenger receptor BI, and ATP-binding cassette transporter G1 (ABCG1) [47]. Furthermore, in a study by Liang et al. [46], it was indicated that *P. gingivalis* induced foam cell formation and CD36 upregulation through NF-κB, and ERK 1/2 pathways and nuclear translocation of p65 [46]. In contrast to the previous study [47], the upregulation of CD36 was merely reported by the exposure to *P. gingivalis*, whereas macrophage exposure to *Escherichia coli* (*E. coli)* and LPS did not exert any significant effect on CD36 [46]. The proposed mechanism by which the CD36 induces lipid accumulation is by the activation of the nuclear hormone receptor of peroxisome proliferator-activated receptor–gamma (PPAR-γ) by oxLDL [53, 54]. The activation of PPAR-γ positively affects the CD36 expression, which will accelerate the oxLDL internalization by foam cells. The role of PPAR–γ in foam cell formation is further confirmed by Luo et al. [55]. They [55] have indicated that the activation of the PPAR signaling pathway is an important factor in promoting adipogenic differentiation genes and the resultant intracellular lipid accumulation which makes it an important factor in foam cell formation.

As mentioned earlier, impaired lipid transportation is one of the main mechanisms by which *P. gingivalis* could induce foam cell formation [46, 47]. In this regard, Yang et al. [41], have reported that *P. gingivalis* can enhance lysosomal integral membrane protein 2 (LIMP2) expression levels in macrophages through NF–κB and JNK pathways. Moreover, it was shown that LIMP2 knockdown can contribute to enhanced cholesterol efflux and decreased foam cell formation [41]. It is postulated that the interaction of LIMP2 and caveolin–1 would explain part of the underlying mechanism of foam cell formation [41]. In addition, *P. gingivalis* inhibits the ABCA1 and ABCG1 cooperation which mediates the cholesterol efflux; therefore, cholesterol will not be removed from the lysosomes, and consequently results in aggravated intracellular cholesterol [41].

Moreover, in a study by Gupta et al. [44], it was shown that *P. gingivalis* and matrix stiffness, which is induced by *P. gingivalis* LPS, can enhance the expression of a Ca^2+^ influx channel called Transient receptor potential channel of the vanilloid subfamily 4 (TRPV4). The knockdown of this mechanosensitive receptor is shown to have inhibitory effects on *P. gingivalis* LPS–induced foam cell formation [44]. The authors [44] reported that TRPV4 mediates oxidized – LDL internalization but not its cell surface binding in macrophages. It would seem that *P. gingivalis* LPS is able to enhance Ca^2+^ influx in macrophages by upregulating the expression of TRPV4. On the other hand, TRPV4 in endothelial cells is shown to have athero-protective effects by inhibiting monocytes adhesion to endothelial cells and activation of endothelial NO synthase (eNOS) [49]. In contrast, TRPV4 channels insufficiency would lead to reduced foam cell formation, endothelial impairment, and vascular disease [51, 56–58].

Another mechanism by which *P. gingivalis* prompts foam cell formations is through fimbria [37]. The fimbria–deficient *P. gingivalis* is shown to be unable to adhere to and invade cells [59–61] and induce alveolar bone loss in the oral cavity [62]. Concerning their effects on macrophages, fimbria–deficient *P. gingivalis* are unable to promote foam cell formation and macrophage invasion [37]. The fimbria of *P. gingivalis* is proven to enhance the proinflammatory cytokines in macrophages [37]. In this regard, CD18 is shown to have an important role in signal transduction [63, 64]. The *P. gingivalis* minor fimbria are proven to enhance proinflammatory cytokines, including interleukin-6 (IL–6) through CD14 and toll–like receptor 2 (TLR–2) [65, 66]. The *P. gingivalis* major fimbria are also shown to have a significant role in foam cell formation [37]. The *P. gingivalis* fimbria’s interaction with β2–integrin of macrophage is reported to be important in the *P. gingivalis* internalization [64]. The fimbria–deficient *P. gingivalis* have exerted reduced catalytic activity compared to the wild–type *P. gingivalis* due to gingipain activity [67].

In addition, TLRs are suggested to have an imperative role in inflammatory response against *P. gingivalis* [50]. These molecules support innate immune recognition of pathogen–associated molecular patterns, such as lipopolysaccharides (TLR4), lipoproteins (TLR2), etc. [68]. In order to ignite the intracellular cascade by TLRs, myeloid differentiation factor 88 (MyD88), TRIF (TLR–domain–containing adaptor–inducing interferon–β), and so forth should be recruited [69]. Based on the results of Shaik–Dasthagirisaheb et al. [50], MyD88 and lps2 (the gene of TRIF) play a significant role in foam cell induction by *P. gingivalis*. Moreover, it was shown that heat–killed *P. gingivalis* and alive *P. gingivalis* exert the same ability in inducing foam cell formation [50]. This finding suggests the possible role of LPS of *P. gingivalis* in foam cell formation. Moreover, it was shown that the presence of *P. gingivalis* and *P. gingivalis* + LDL can significantly enhance the TNF–α and IL– 6 productions by macrophages [50]. However, the combination of LDL and *P. gingivalis* seemed to reduce the cytokine release compared to *P. gingivalis* alone [50].

Another important factor in inducing foam cell formation by *P. gingivalis* is the multiplicity of infection (MOI) of bacteria. Shaik–Dasthagirisaheb et al. [50], have shown that the higher the MOI of *P. gingivalis*, the higher rate of foam cell formation. In addition, in another study by Shaik–Dasthagirisaheb et al., it was reported that *P. gingivalis* and *Chlamydia pneumoniae* (*C. pneumonia)* can induce foam cell formation in bone marrow–derived macrophages (BMDMs) in MOI of 100 and 10, respectively [43]. The exposure of these pathogens to LDL–treated BMDM elevated the tumor necrosis factor-α (TNF–α), IL–6, and IL–1β (this factor was exclusively enhanced by *C. pneumonia*) [43]. Similarly, as mentioned in the previous study, LDL downregulates cytokines secretion [50]. The analysis of the cytokine release profile indicated that the cytokine response is not identical for all the pathogens that can cause foam cell formation [43].

Another mechanism concerning the possible effect of *P. gingivalis* on foam cell formation and atherosclerosis is through the suppression of heme oxygenase–1 (HO–1) [47]. This enzyme plays an imperative role in the prevention of vascular inflammation through anti–oxidant, anti–inflammatory, anti–proliferative, anti–apoptotic, and immunomodulatory effects which have shown athero–protective effects [70]. According to Li et al. [47], the HO–1 knockdown results in CD36 and ABCA1 downregulation, and activation of c-Jun-AP/1. In other words, inhibition of HO-1 exacerbates the effect of *P. gingivalis* LPS and aggravates the intracellular lipid content [47].

Aside from the mechanisms mentioned above, another pathway that the authors of this review believe to play a role in this regard is the metabolic changes in macrophages due to *P. gingivalis* infection [71, 72]. *P. gingivalis* can increase the activity of the lactic acid cycle while decreasing oxidative phosphorylation [71, 72]. This process can enhance cellular lactic acid storage, decline mitochondrial oxygen usage, and increase the load of ROS [71, 72]. The enhancement of cellular ROS in macrophages results in a higher rate of lipid oxidation and oxLDL production [71, 72].

This systematic review highlights the importance of periodontal pathogens, especially *P. gingivalis*, in the progression of atherosclerosis which can have significant clinical implications in the long term. Concerning the level of risk of bias, the studies have shown moderate to high levels. Therefore, for future studies in this field, we suggest further well-designed in-vitro studies with low risk of bias and equal in-vitro settings. This would aid future systematic reviews to be able to estimate a predictable correlation between the infection of *P. gingivalis* and the rate of foam cell formation. Moreover, grey literature was not assessed in the current systematic review. Thus, we recommend adding the grey literature in the search strategy of future systematic reviews to provide comprehensive data for screening.

Moreover, according to the compiled outcomes of all the included studies it might be possible to suggest that periodontal treatment procedures could avoid the process of foam cell formation in the arterial intima layer by minimizing the population of *P. gingivalis* in the subgingival plaque area [9, 73]. In this regard, the adjunct application of inflammation-modulatory agents, including nutraceutical agents could also be a treatment option to lessen the severity of the periodontal disease as well [74–77]. In this regard, we strongly recommend further in-vitro, in-vivo, and clinical experiments to assess the reliability of our hypothesis. It is also important to note that the connection of periodontal diseases with atherosclerosis cannot be solely explained based on the effect of *P. gingivalis* on foam cell formation. When interpreting these results, one should be aware that the connection between periodontitis and atherosclerosis is reported to be mediated through various mechanisms, including bacterial species, miRNAs, and so on. Concerning the types of periodontal microbiota *P. gingivalis* is indicated to be the most significant one [73, 78]. On the other hand, the release of certain types of miRNAs into the gingival crevicular fluid can lead to higher susceptibility to cardiovascular diseases by altering gene expression in cardiovascular tissues [79]. Although foam cell formation is central to atherosclerosis, it doesn’t involve the whole mechanism of pathogenesis and development of atherosclerosis [80]. Therefore, we can conclude that *P. gingivalis* could contribute to the process of foam cell formation and this periodontal pathogen may enhance the likelihood of developing atherosclerosis.

## Conclusion

Our study has shed light on the mechanisms by which *P. gingivalis* can promote the process of foam cell formation. Based on the gathered evidence, *P. gingivalis* affects the macrophages’ environment, their gene expression patterns, and cellular mechanisms through which macrophages enhance their lipid uptake and transform into foam cells. The changes in the environment include the effect of *P. gingivalis* on endothelial cells to gather more monocytes to the site and changes in the mechanical and biological properties of the ECM. Moreover, the changes in the gene expression patterns in macrophages can outweigh the equilibrium of lipid transportation into more lipid influx and less lipid efflux. Besides, *P. gingivalis* leads the process of foam cell formation through various cellular mechanisms, including pro-inflammatory cytokines secretion, modification of LDL and HDL, ignition of various cellular signaling pathways, and cell receptor activities. All these processes are ascribed to four marked characteristics in *P. gingivalis*, including MOI of *P. gingivalis*, *P. gingivalis* LPS, *P. gingivalis* major fimbria, and *P. gingivalis* OMV which have demonstrated significant impacts. Since the risk of bias of the included studies in this systematic review are moderate to high, future well-organized studies are required to further confirm the current results.

## Data Availability

The datasets used and/or analyzed during the current study available from the corresponding author on reasonable request.
